# A cytosolic oxidation–reduction cycle in plant leaves

**DOI:** 10.1093/jxb/erab128

**Published:** 2021-03-19

**Authors:** Thomas Wieloch

**Affiliations:** 1 Department of Medical Biochemistry and Biophysics, Umeå University, 901 87 Umeå, Sweden; 2 MPI of Molecular Plant Physiology, Germany

**Keywords:** Cytosolic oxidation–reduction cycle, energy metabolism, energy status, futile carbon cycling, glyceraldehyde-3-phosphate dehydrogenase, NADPH, oxidative stress, primary carbon metabolism, reactive oxygen species, redox status

## Abstract

The viewpoint proposes a carbon-neutral biochemical cycle in the cytosol of plant leaves that is up-regulated by reactive oxygen species. Cycling provides NADPH and dissipates energy to counteract oxidative stress.

**Leaf cytosol contains non-phosphorylating and phosphorylating glyceraldehyde-3-phosphate dehydrogenase (np-GAPDH and p-GAPDH, respectively). From the viewpoint of carbon metabolism, np-GAPDH is redundant. However, mutants lacking np-GAPDH show significant metabolic adjustments and decreased growth, suggesting that np-GAPDH has central functions in plant metabolism. Here, I propose a cytosolic oxidation–reduction cycle. In its forward direction, np-GAPDH supplies NADPH. In the reverse direction, phosphoglycerate kinase and p-GAPDH consume ATP and NADH. Thus, the cytosolic oxidation–reduction cycle may constitute a central hub in energy metabolism.**

In the light, NADPH is primarily synthesized in chloroplasts. However, NADPH is highly compartmentalized (Heber and Santarius, 1965). Chloroplastic NAD^+^ carrier proteins in reconstituted systems show low affinities for this reductant (Palmieri *et al.*, 2009). Thus, NADPH export to the cytosol is not straightforward.

Currently, it is thought that glucose-6-phosphate dehydrogenase, 6-phosphogluconate dehydrogenase, and NADP^+^-dependent isocitrate dehydrogenase supply cytosolic NADPH (Geigenberger and Fernie, 2014). Additionally, carbon cycling around non-phosphorylating (np)-glyceraldehyde 3-phosphate dehydrogenase (GAPDH) was suggested to provide cytosolic NADPH (Fig. 1; Kelly and Gibbs, 1973*a*, *b*; Scagliarini *et al.*, 1990). In this cycle, (i) dihydroxyacetone phosphate is exported from chloroplasts to the cytosol by the triose phosphate translocator and converted to glyceraldehyde 3-phosphate (GAP) by triose phosphate isomerase, (ii) GAP is oxidized to 3-phosphoglyceric acid (3PGA) and NADP^+^ is reduced to NADPH by np-GAPDH, and (iii) 3PGA is reimported into chloroplasts by the triose phosphate translocator and reduced to dihydroxyacetone phosphate by chloroplastic phosphoglycerate kinase (PGK) and phosphorylating (p)-GAPDH. A similar cycle involving p-GAPDH and PGK in the forward direction was suggested to provide cytosolic NADH and ATP (Fig. 1; Stocking and Larson, 1969). However, Heber and Santarius (1970) questioned its occurrence *in vivo*.

**Fig. 1.**
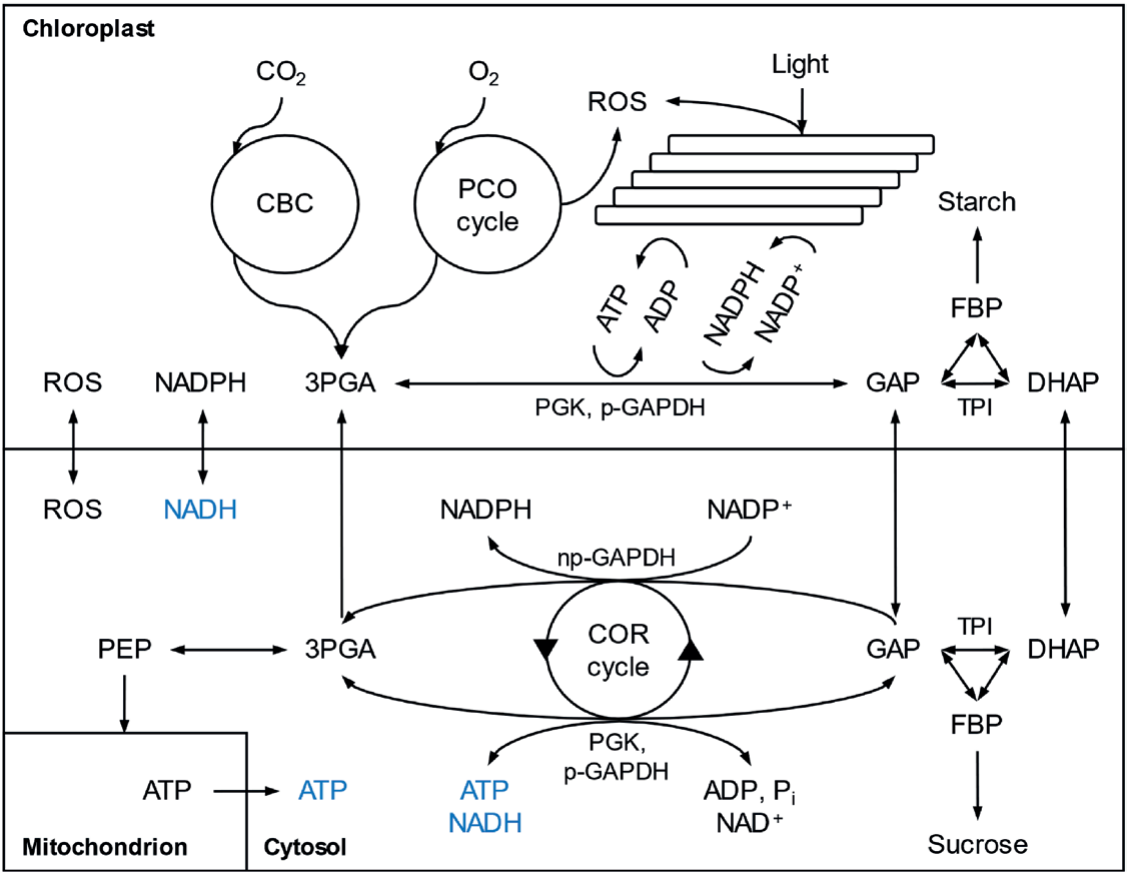
Carbon and energy metabolism in plant leaves. Parts of the PCO cycle reside outside chloroplasts, in peroxisomes and mitochondria; notably, ROS synthesizing glycolate oxidase is peroxisomal. Abbreviations: 3PGA, 3-phosphoglyceric acid; ADP, adenosine diphosphate; ATP, adenosine triphosphate; CBC, Calvin–Benson cycle; COR cycle, cytosolic oxidation–reduction cycle; DHAP, dihydroxyacetone phosphate; FBP, fructose 1,6-bisphosphate; GAP, glyceraldehyde 3-phosphate; np-GAPDH, non-phosphorylating glyceraldehyde-3-phosphate dehydrogenase; PCO, photosynthetic carbon oxidation; PEP, phosphoenolpyruvate; p-GAPDH, phosphorylating glyceraldehyde-3-phosphate dehydrogenase; PGK, phosphoglycerate kinase; P_i_, inorganic phosphate; ROS, reactive oxygen species; TPI, triose phosphate isomerase.

Here, another route supplying cytosolic NADPH is proposed: the cytosolic oxidation–reduction (COR) cycle (Fig. 1). In its forward direction, cytosolic np-GAPDH oxidizes GAP to 3PGA and reduces NADP^+^ to NADPH. In the reverse direction, cytosolic PGK and p-GAPDH reduce 3PGA to GAP, dephosphorylate ATP to ADP and P_i_, and oxidize NADH to NAD^+^. COR cycling was shown to be operational in reconstituted enzyme systems (Serrano *et al.*, 1993; Arutyunov and Muronetz, 2003). Here, the COR cycle is discussed as an actual metabolic route with benefits for plant functioning. Specifically, I address (i) how differences in biochemical properties of np-GAPDH and p-GAPDH may promote COR cycling, (ii) how COR cycle requirements may be met, and (iii) physiological benefits of the COR cycle. My aim is to raise awareness that COR cycling may occur *in vivo* and encourage assessments of its metabolic feasibility, e.g. by flux network modelling (cf. Shameer *et al.*, 2019) and the development of methodology enabling flux quantification such as stable isotope techniques.

## Biochemical properties of GAPDH enzymes promote COR cycling

The cytosol of plant leaves contains two distinct GAPDHs. While np-GAPDH catalyses the irreversible conversion of GAP to 3PGA, p-GAPDH together with PGK catalyses the same reaction in a reversible manner (Fig. 1). Thus, from the viewpoint of carbon metabolism, np-GAPDH is redundant. However, np-GAPDH null mutants of Arabidopsis showed delayed growth compared with wild type (Rius *et al.*, 2006). Additionally, these mutants exhibited 3.5-fold higher transcript levels, 5.8-fold higher mRNA levels, and a 2.5-fold increased activity of p-GAPDH, indicating a compensation effect for the lack of np-GAPDH. Thus, np-GAPDH seems to be required for optimal plant functioning and growth (Rius *et al.*, 2006).

At the sequence level, np-GAPDH and p-GAPDH are entirely unrelated (Habenicht *et al.*, 1994; Michels *et al.*, 1994). I argue that a comparison of differences in enzyme properties may point to the physiological necessity for np-GAPDH.

### GAPDH energetics, substrate affinity, and activity

Conversion of GAP to 3PGA by np-GAPDH is energetically favourable over conversion by p-GAPDH and PGK (Δ*G*^0^′=−22.1 versus −13.3 kcal mol^−1^, respectively). Additionally, np-GAPDH from various sources has an ~10-fold higher affinity for GAP than p-GAPDH (*K*_m_ of np-GAPDH between 17 and 40 µM, *K*_m_ of p-GAPDH between 239 and 400 µM; Rosenberg and Arnon, 1955; Kelly and Gibbs, 1973*b*; Duggleby and Dennis, 1974; Speranza and Gozzer, 1978; Iglesias and Losada, 1988; Scagliarini *et al.*, 1990). Furthermore, np-GAPDH reportedly exceeded the activity of p-GAPDH in a cell-free extract from *Pisum sativum* shoots at *in vivo* levels of GAP and reductants (Kelly and Gibbs, 1973*b*). Since np-GAPDH exhibits a higher affinity for GAP, Flügge and Heldt (1984) hypothesized cytosolic NADPH synthesis by np-GAPDH may be prioritized over NADH and ATP synthesis by p-GAPDH and PGK. I argue that growth delays and substantial biochemical compensation effects in np-GAPDH null mutants (Rius *et al.*, 2006) suggest a significant contribution of np-GAPDH to catalysing GAP to 3PGA conversions.

Reported concentrations of GAP and 3PGA in suspension-cultured cells of *Catharanthus roseus* correspond to equilibrium conditions around p-GAPDH and PGK (Kubota and Ashihara, 1990) even though np-GAPDH works against this equilibrium. Thus, p-GAPDH and PGK may readjust their disturbed equilibrium by more frequently catalysing the reverse reaction (3PGA to GAP) than the forward reaction (GAP to 3PGA), which results in COR cycling.

### Activation of np-GAPDH by reactive oxygen species

In natural settings, the cellular redox balance is regularly disturbed, e.g. by low chloroplastic CO_2_ concentrations (*C*_c_) due to drought. Low *C*_c_ promotes an energy excess in chloroplasts due to decreased consumption of ATP and NADPH by the Calvin–Benson cycle but continued electron input by light-harvesting complexes (Wilhelm and Selmar, 2011). The resulting lack of electron acceptors promotes the generation of reactive oxygen species (ROS) especially superoxide and H_2_O_2_. Additionally, low *C*_c_ promotes photorespiration and the generation of the photorespiratory side product H_2_O_2_. Under low *C*_c_, photorespiration was estimated to generate >70% of all H_2_O_2_ (Noctor *et al.*, 2002). Thus, low *C*_c_ promotes oxidative stress leading to increased oxidation of cytosolic NADPH by antioxidant systems. *In vivo*, the NADPH concentration proposedly exerts primary control over np-GAPDH activity with decreasing concentrations activating np-GAPDH (Kelly and Gibbs, 1973*b*; Iglesias and Losada, 1988; Scagliarini *et al.*, 1990). Thus, decreasing NADPH due to oxidative stress at low *C*_c_ causes np-GAPDH activation and promotes the GAP to 3PGA forward reaction of COR cycling.

### Inhibition of p-GAPDH by reactive oxygen species

np-GAPDH is 63 times less susceptible to inhibition by ROS than p-GAPDH, with H_2_O_2_ being a particularly potent inhibitor of p-GAPDH (Piattoni *et al.*, 2013). Rising H_2_O_2_ levels are believed to progressively inhibit the reversible p-GAPDH (Hancock *et al.*, 2005; Bedhomme *et al.*, 2012; Piattoni *et al.*, 2013). This is corroborated by reported increases of glycolytic downstream metabolites including 3PGA under oxidative conditions (Baxter *et al.*, 2007; Lehmann et al., 2009, 2012; Rabara *et al.*, 2017). At PGK and p-GAPDH, increased 3PGA levels would promote the reverse reaction (3PGA to GAP). Thus, the COR cycling flux mode may be promoted under oxidative conditions.

Interestingly, inactive p-GAPDH functions as transcription factor triggering the induction of genes encoding antioxidant enzymes. For more information on GAPDH regulation including moonlighting functions see Scheibe *et al.* (2019) and references therein.

## Requirements of the COR cycle

### Reverse reactions require ATP and NADH

COR cycling reverse reactions (3PGA to GAP) catalysed by PGK and p-GAPDH require cytosolic ATP and NADH (Fig. 1). This requirement may increase with oxidative stress, e.g. due to low *C*_c_ (see above). Much of the cytosolic ATP is believed to derive from the mitochondrial oxidation of photorespiratory glycine (Shameer *et al.*, 2019). Low *C*_c_ promotes photorespiratory glycine oxidation. Moreover, low *C*_c_ is often associated with excess amounts of NADPH in chloroplasts (see above). Excess NADPH in chloroplasts is shuttled out to the cytosol by the malate valve as NADH. Flux through the valve is regulated strictly by the activity of chloroplastic malate dehydrogenase, which increases with [NADPH]/[NADP^+^] ratios (Fridlyand *et al.*, 1998). Under normal conditions, flux is low, yet kinetic modelling predicts much enhanced rates at high [NADPH]/[NADP^+^] ratios, e.g. due to low *C*_c_ under drought (Fridlyand *et al.*, 1998). Drought was shown to cause significantly increased activity of chloroplastic malate dehydrogenase in *Triticum aestivum* particularly under high light and moderate ambient CO_2_ levels of 350 ppm (Biehler *et al.*, 1996). Thus, export of ATP and NADH to the cytosol is generally feasible. Mechanisms increasing ATP and NADH supply coincide with increased NADPH demands from COR cycling for ROS scavenging.

### Retaining activity of p-GAPDH under oxidative conditions

Reactive oxygen species inhibit p-GAPDH (see above). If all p-GAPDH were in its oxidized inactive state, COR cycling would halt. Hence, cycling requires retained p-GAPDH activity. Bedhomme *et al.* (2012) proposed cytosolic glutaredoxin and thioredoxin-based mechanisms reversing oxidative deactivation of p-GAPDH. Thus, part of the p-GAPDH population is likely always active.

## Benefits of COR cycling for plant functioning

COR cycling involves only three cytosolic enzymes and is carbon neutral. It requires no net carbon input, causes no net carbon loss, and does not produce carbon products that need to be consumed by other processes. Hence, COR cycling is independent of other parts of carbon metabolism as well as transmembrane transport of triose or pentose phosphates. This provides flexibility to its functions.

*In vivo*, NADPH concentration is believed to exert primary control over np-GAPDH activity (see above). Thus, COR cycling may help to maintain NADPH concentrations at high levels to steadily support all NADPH-consuming processes in the cytosol.

Optimal plant functioning requires a well-balanced energy supply versus consumption. Under most conditions, NADPH and ATP supply exceed metabolic demands, and several processes dissipating excess energy have been proposed (Wilhelm and Selmar, 2011). Export of reductant and ATP to the cytosol can remove excess energy from chloroplasts and mitochondria, respectively. COR cycling can dissipate this energy because each turn produces one molecule NADPH but consumes one molecule of ATP and NADH. Thus, COR cycling may counteract the generation of ROS. Additionally, NADPH from COR cycling may fuel cytosolic antioxidant systems and thus support ROS scavenging.

## Abbreviations

3PGA: 3-phosphoglyceric acid
COR: cytosolic oxidation–reduction
GAP: glyceraldehyde 3-phosphate
np-GAPDH: non-phosphorylating glyceraldehyde-3-phosphate dehydrogenase
p-GAPDH: phosphorylating glyceraldehyde-3-phosphate dehydrogenase
PGK: phosphoglycerate kinase
ROS: reactive oxygen species

## References

[CIT0001] Arutyunov DY , Muronetz VI. 2003. The activation of glycolysis performed by the non-phosphorylating glyceraldehyde-3-phosphate dehydrogenase in the model system. Biochemical and Biophysical Research Communications 300, 149–154. 12480534 10.1016/s0006-291x(02)02802-4

[CIT0002] Baxter CJ , Redestig H, Schauer N, Repsilber D, Patil KR, Nielsen J, Selbig J, Liu J, Fernie AR, Sweetlove LJ. 2007. The metabolic response of heterotrophic Arabidopsis cells to oxidative stress. Plant Physiology 143, 312–325. 17122072 10.1104/pp.106.090431 PMC1761969

[CIT0003] Bedhomme M , Adamo M, Marchand CH, Couturier J, Rouhier N, Lemaire SD, Zaffagnini M, Trost P. 2012. Glutathionylation of cytosolic glyceraldehyde-3-phosphate dehydrogenase from the model plant *Arabidopsis thaliana* is reversed by both glutaredoxins and thioredoxins *in vitro*. The Biochemical Journal 445, 337–347. 22607208 10.1042/BJ20120505

[CIT0004] Biehler K , Migge A, Fock HP. 1996. The role of malate dehydrogenase in dissipating excess energy under water stress in two wheat species. Photosynthetica 32, 431–438.

[CIT0005] Duggleby RG , Dennis DT. 1974. Nicotinamide adenine dinucleotide-specific glyceraldehyde 3-phosphate dehydrogenase from *Pisum sativum*: assay and steady state kinetics. Journal of Biological Chemistry 249, 167–174. 4358627

[CIT0006] Flügge UI , Heldt HW. 1984. The phosphate-triose phosphate-phosphoglycerate translocator of the chloroplast. Trends in Biochemical Sciences 9, 530–533.

[CIT0007] Fridlyand LE , Backhausen JE, Scheibe R. 1998. Flux control of the malate valve in leaf cells. Archives of Biochemistry and Biophysics 349, 290–298. 9448717 10.1006/abbi.1997.0482

[CIT0008] Geigenberger P , Fernie AR. 2014. Metabolic control of redox and redox control of metabolism in plants. Antioxidants & Redox Signaling 21, 1389–1421. 24960279 10.1089/ars.2014.6018 PMC4158967

[CIT0009] Habenicht A , Hellman U, Cerff R. 1994. Non-phosphorylating GAPDH of higher plants is a member of the aldehyde dehydrogenase superfamily with no sequence homology to phosphorylating GAPDH. Journal of Molecular Biology 237, 165–171. 7545914 10.1006/jmbi.1994.1217

[CIT0010] Hancock JT , Henson D, Nyirenda M, Desikan R, Harrison J, Lewis M, Hughes J, Neill SJ. 2005. Proteomic identification of glyceraldehyde 3-phosphate dehydrogenase as an inhibitory target of hydrogen peroxide in *Arabidopsis*. Plant Physiology and Biochemistry 43, 828–835. 16289945 10.1016/j.plaphy.2005.07.012

[CIT0011] Heber UW , Santarius KA. 1965. Compartmentation and reduction of pyridine nucleotides in relation to photosynthesis. Biochimica et Biophysica Acta 109, 390–408. 4379647 10.1016/0926-6585(65)90166-4

[CIT0012] Heber U , Santarius KA. 1970. Direct and indirect transfer of ATP and ADP across the chloroplast envelope. Zeitschrift fur Naturforschung 25, 718–728. 4394211 10.1515/znb-1970-0714

[CIT0013] Iglesias AA , Losada M. 1988. Purification and kinetic and structural properties of spinach leaf NADP-dependent nonphosphorylating glyceraldehyde-3-phosphate dehydrogenase. Archives of Biochemistry and Biophysics 260, 830–840. 3341766 10.1016/0003-9861(88)90514-0

[CIT0014] Kelly GJ , Gibbs M. 1973 *a*. A mechanism for the indirect transfer of photosynthetically reduced nicotinamide adenine dinucleotide phosphate from chloroplasts to the cytoplasm. Plant Physiology 52, 674–676. 16658629 10.1104/pp.52.6.674 PMC366570

[CIT0015] Kelly GJ , Gibbs M. 1973 *b*. Nonreversible D-glyceraldehyde 3-phosphate dehydrogenase of plant tissues. Plant Physiology 52, 111–118. 16658509 10.1104/pp.52.2.111 PMC366450

[CIT0016] Kubota K , Ashihara H. 1990. Identification of non-equilibrium glycolytic reactions in suspension-cultured plant cells. Biochimica et Biophysica Acta 1036, 138–142. 2223831 10.1016/0304-4165(90)90025-r

[CIT0017] Lehmann M , Laxa M, Sweetlove LJ, Fernie AR, Obata T. 2012. Metabolic recovery of *Arabidopsis thaliana* roots following cessation of oxidative stress. Metabolomics 8, 143–153. 22279429 10.1007/s11306-011-0296-1 PMC3258409

[CIT0018] Lehmann M , Schwarzländer M, Obata T, et al. 2009. The metabolic response of *Arabidopsis* roots to oxidative stress is distinct from that of heterotrophic cells in culture and highlights a complex relationship between the levels of transcripts, metabolites, and flux. Molecular Plant 2, 390–406. 19825624 10.1093/mp/ssn080

[CIT0019] Michels S , Scagliarini S, Della Seta F, Carles C, Riva M, Trost P, Branlant G. 1994. Arguments against a close relationship between non-phosphorylating and phosphorylating glyceraldehyde-3-phosphate dehydrogenases. FEBS Letters 339, 97–100. 8313985 10.1016/0014-5793(94)80393-5

[CIT0020] Noctor G , Veljovic-Jovanovic S, Driscoll S, Novitskaya L, Foyer CH. 2002. Drought and oxidative load in the leaves of C_3_ plants: a predominant role for photorespiration? Annals of Botany 89, 841–850. 12102510 10.1093/aob/mcf096 PMC4233806

[CIT0021] Palmieri F , Rieder B, Ventrella A, et al. 2009. Molecular identification and functional characterization of *Arabidopsis thaliana* mitochondrial and chloroplastic NAD^+^ carrier proteins. The Journal of Biological Chemistry 284, 31249–31259. 19745225 10.1074/jbc.M109.041830 PMC2781523

[CIT0022] Piattoni CV , Guerrero SA, Iglesias AA. 2013. A differential redox regulation of the pathways metabolizing glyceraldehyde-3-phosphate tunes the production of reducing power in the cytosol of plant cells. International Journal of Molecular Sciences 14, 8073–8092. 23584025 10.3390/ijms14048073 PMC3645732

[CIT0023] Rabara RC , Tripathi P, Rushton PJ. 2017. Comparative metabolome profile between tobacco and soybean grown under water-stressed conditions. BioMed Research International 2017, 3065251. 28127554 10.1155/2017/3065251 PMC5239840

[CIT0024] Rius SP , Casati P, Iglesias AA, Gomez-Casati DF. 2006. Characterization of an *Arabidopsis thaliana* mutant lacking a cytosolic non-phosphorylating glyceraldehyde-3-phosphate dehydrogenase. Plant Molecular Biology 61, 945–957. 16927206 10.1007/s11103-006-0060-5

[CIT0025] Rosenberg LL , Arnon DI. 1955. The preparation and properties of a new glyceraldehyde-3-phosphate dehydrogenase from photosynthetic tissues. The Journal of Biological Chemistry 217, 361–371. 13271400

[CIT0026] Scagliarini S , Trost P, Valenti V, Pupillo P. 1990. Glyceraldehyde 3-phosphate:NADP reductase of spinach leaves: steady state kinetics and effect of inhibitors. Plant Physiology 94, 1337–1344. 16667838 10.1104/pp.94.3.1337 PMC1077383

[CIT0027] Scheibe R . 2019. Maintaining homeostasis by controlled alternatives for energy distribution in plant cells under changing conditions of supply and demand. Photosynthesis Research 139, 81–91. 30203365 10.1007/s11120-018-0583-z PMC6373317

[CIT0028] Serrano A , Mateos MI, Losada M. 1993. ATP-driven transhydrogenation and ionization of water in a reconstituted glyceraldehyde-3-phosphate dehydrogenases (phosphorylating and non-phosphorylating) model system. Biochemical and Biophysical Research Communications 197, 1348–1356. 8280152 10.1006/bbrc.1993.2625

[CIT0029] Shameer S , Ratcliffe RG, Sweetlove LJ. 2019. Leaf energy balance requires mitochondrial respiration and export of chloroplast NADPH in the light. Plant Physiology 180, 1947–1961. 31213510 10.1104/pp.19.00624 PMC6670072

[CIT0030] Speranza ML , Gozzer C. 1978. Purification and properties of NAD^+^-dependent glyceraldehyde-3-phosphate dehydrogenase from spinach leaves. Biochimica et Biophysica Acta 522, 32–42. 202324 10.1016/0005-2744(78)90319-4

[CIT0031] Stocking CR , Larson S. 1969. A chloroplast cytoplasmic shuttle and the reduction of extraplastid NAD. Biochemical and Biophysical Research Communications 37, 278–282. 4390456 10.1016/0006-291x(69)90731-1

[CIT0032] Wilhelm C , Selmar D. 2011. Energy dissipation is an essential mechanism to sustain the viability of plants: the physiological limits of improved photosynthesis. Journal of Plant Physiology 168, 79–87. 20800930 10.1016/j.jplph.2010.07.012

